# Integrating Transcriptomic and Proteomic Data: IL‐27B as a Key Protein in the Development of Septic Cardiomyopathy—A Retrospective Study

**DOI:** 10.1002/iid3.70207

**Published:** 2025-05-21

**Authors:** Yifeng Mao, Qingqing Chen, Yongpo Jiang, Xijiang Zhang, Qin Si, Panpan Xu, Zhongheng Zhang, Cheng Zheng, Ronghai Lin

**Affiliations:** ^1^ Department of Critical Care Medicine Municipal Hospital Affiliated to Taizhou University Taizhou ZheJiang Province China; ^2^ Department of Critical Care Medicine Taizhou Hospital of Zhejiang Province Affiliated to Wenzhou Medical University Linhai Zhejiang Province China; ^3^ Department of Neurorehabilitation Center Taizhou Rehabilitation Hospital, Taizhou Enze Medical Center (Group) Taizhou Zhejiang Province China; ^4^ Department of Emergency Medicine, Sir Run Run Shaw Hospital Zhejiang University School of Medicine Hangzhou Zhejiang Province China

**Keywords:** multiomics, proteomics, septic cardiomyopathy, transcriptomics

## Abstract

**Background:**

Septic cardiomyopathy (SCM) is a potentially fatal complication of sepsis. In this study, transcriptomic and proteomic analyzes of serum samples from sepsis patients were conducted to uncover the underlying pathological mechanisms and identify potential therapeutic targets for SCM.

**Methods:**

This retrospective, dual‐center study investigated the progression of sepsis to SCM in patients admitted to intensive care units. A total of 50 patients were enrolled and divided into two groups: sepsis with cardiomyopathy (25 cases) and sepsis without cardiomyopathy (25 cases). Co‐expression network analysis was employed to elucidate the biological significance of differentially expressed proteins. By integrating proteomic and transcriptomic data, molecular networks were constructed to visualize interactions among key molecules, aiming to enhance data interpretation and support the study's findings.

**Results:**

Proteomic analysis identified 216 differentially expressed proteins (Fold change > 1.5, *p*‐value < 0.05) between the two groups. Transcriptomic analysis revealed two proteins, including Interleukin‐27 subunit beta (IL‐27B) and carbonic anhydrase, co‐downregulated in patients with septic cardiomyopathy. IL‐27B was associated with the immune response, and Kyoto Encyclopedia of Genes and Genomes (KEGG) pathway enrichment analysis indicated its involvement in the cytokine‐cytokine receptor interaction signaling pathway.

**Conclusion:**

Comprehensive integrated transcriptomic and proteomic analyzes identified significant changes in protein expression associated with SCM, primarily associated with inflammation‐related pathways and amino acid metabolism. These findings provide new insights into the pathological mechanisms of SCM and highlight potential therapeutic targets for its treatment.

**Trial Registration:**

The Clinical Research Ethics Committee of Taizhou Hospital of Zhejiang Province affiliated to Wenzhou Medical University approved this study, and written informed consent was given by all patients or their legal representatives. (NO.K20201110).

## 1 Introduction

Sepsis is a severe condition characterized by potentially fatal organ dysfunction resulting from dysregulated immune responses to infection. It remains the leading cause of mortality in intensive care units (ICUs), affecting 20%–30% of ICU admissions [1, 2, 3]. A recent study reported an incidence of 58 sepsis cases per 100,000 persons annually in ICUs, with a predischarge mortality rate of 41.9% [4]. Sepsis‐related immune dysregulation can lead to severe organ dysfunction, with septic cardiomyopathy (SCM) associated with the highest mortality rates. Septic cardiomyopathy (SCM), which involves impaired myocardial contractile and diastolic function on both sides of the heart, affects approximately 13.8% of patients with sepsis and septic shock [5]. Given the lack of effective treatments and the high mortality rates, SCM is considered one of the most significant contributors to sepsis‐related deaths [6, 7].

Early detection of myocardial dysfunction is vital for optimizing sepsis management; however, traditional metrics such as ejection fraction (EF) are insufficient for evaluating this dysfunction. Thus, there is a pressing need for molecular and subcellular analyzes to better understand the influence of immunometabolic and neuroendocrine factors on cardiomyocytes, fibroblasts, and endothelial cells [8]. A study reported that approximately 50% of patients with sepsis exhibited reduced left ventricular ejection fraction (LVEF), accompanied by increases in both end‐systolic volume (ESV) and end‐diastolic volume (EDV) [9]. However, the definition of SCM varies, with some studies describing it as a reversible reduction in bilateral ventricular EF, ventricular dilation, and decreased responsiveness to fluid resuscitation and catecholamines [10, 11]. SCM has also been characterized as sepsis‐induced intramyocardial systolic and diastolic dysfunction affecting both the left and right ventricles [12, 13]. Despite extensive research, the pathophysiology of SCM remains poorly understood, and no specific pharmacological treatments exist to reverse sepsis‐induced myocardial dysfunction, leaving supportive care as the primary treatment option [14]. Studies have suggested that cytokines such as TNF‐α, IL‐1, and IL‐6, known as myocardial inhibitors, could serve as potential therapeutic targets for SCM [15]. Therefore, investigating sepsis‐related proteins is important for advancing the diagnosis and treatment of SCM.

The central dogma of molecular biology posits that genetic information flows from Deoxyribonucleic Acid (DNA) to messenger Ribonucleic Acid (mRNA) to proteins [16]. However, gene expression regulation occurs at multiple levels, leading to limited concordance between mRNA and protein expression. Thus, integrating transcriptomic and proteomic analyzes can provide a more comprehensive understanding of gene expression dynamics [17]. Studies have shown that only 27%–40% of gene expression consistency is observed between mRNA and protein levels, highlighting the importance of multiomics approaches to study gene expression [18, 19, 20]. For example, Petersen et al. used transcriptomic and proteomic analyzes to determine that the hydra‐head rebirth process is facilitated by the induction of Wnt and TGF‐β‐related signaling responses while also revealing no role of neurons, stem cells, or derivatives in this process [21] while Wang et al. employed similar techniques to study metabolic regulation in *Bacillus thuringiensis* and the mechanisms governing hemispora crystal formation [22].

To date, few studies have focused on the proteomic characterization of sepsis, and proteomic‐based diagnostic tests for sepsis are still lacking. Furthermore, no specific protein targets for SCM have been identified, prompting the present study to address this gap. The present study employed transcriptomic and proteomic analyzes to gain multi‐omics insights into the molecular changes underlying the development of SCM, providing a foundation for future research on the pathogenesis and diagnosis of this life‐threatening condition.

## 2 Methods

### 2.1 Study Design

This is a retrospective study based on septic patients admitted to Municipal hospital affiliated to Taizhou University and Taizhou Hospital of Zhejiang Province affiliated to Wenzhou Medical University in 2021. Clinical data and follow‐up information were collected from electronic medical records and case report forms, with the most recent follow‐up on November 5, 2022. Sepsis was diagnosed based on an acute change of two or more points in the total sequential organ failure assessment (SOFA) score attributable to infection [2]. SCM was diagnosed using the following criteria:

1. EF  < 50% during the observation period;
2. A reduction of at least 10% from baseline in LVEF;
3. Abnormal ventricular wall motion, characterized by decreased full‐wall or partial‐wall motion [5].

Patients without SCM were designated as the non‐SCM group, while those with SCM comprised the experimental group. SCM‐associated proteins were identified through differential expression and co‐expression network analysis. Transcriptomic and proteomic data were integrated to investigate the pathogenesis of SCM. The study was approved by the Clinical Research Ethics Committee of Taizhou Hospital of Zhejiang Province affiliated to Wenzhou Medical University, and written informed consent was obtained from all participants or their legal representatives.

### 2.2 Sample Collection and Preparation

Blood samples were collected from patients diagnosed with sepsis upon ICU admission using serological separation tubes. After allowing to coagulate at room temperature for 1 h, samples were centrifuged (30*g*, 10 min), and the harvested sera were stored at −80°C until Liquid Chromatography‐Tandem Mass Spectrometry (LC‐MS/MS) analysis.

### 2.3 Transcriptomic Analysis

The serum samples were lyophilized. Genetic analysis of sera from SCM and non‐SCM patients was conducted using *Homo sapiens* genomic data (human: NCBI_GCF_000001405.39_GRCh38.p13) as the reference.

### 2.4 Peptide Isolation

An LC‐20AD liquid phase system (Shimadzu, Japan) with a Gemini C18 column (20 cm × 180 × 5 μm) was used for separating mixed 20 μg protein samples. Peptides in mobile phase A (5% ACN, pH 9.8) were injected, followed by gradient elution using 2% mobile phase B (95% ACN, pH 9.8) for 7 min, 2%‒7% B for 3 min, 7%‒25% B for 27 min, 25%‒60% B for 2 min, 60%‒80% B for 1 min, B for 3 min, 80%‒2% B for 2 min, 2% B equilibration for 5 min. Elution peaks were recorded at 214 nm, collecting one sample every 3.15 min, finally yielding 10 fractions generated by combining samples according to chromatographic elution peak maps.

### 2.5 High pH RP Separation

Equal amounts of peptides (20 μg) from individual samples were mixed, and the resultant 20 μg sample was added to 2 mL of mobile phase A (as above) and injected into an LC‐20AB liquid phase system (Shimadzu) with a Gemini C18 column (4.6 × 125 mm × 5 μm). Elution was performed at a 1 mL/min flow rate using the following settings: 5% mobile phase B (as above) for 10 min, 5%‒35% B for 40 min, 35%‒95% B for 1 min, B for 3 min, and 5% B equilibration for 10 min. Elution was monitored at 214 nm, and fractions were collected and combined according to chromatographic elution peaks. The samples were then lyophilized.

### 2.6 Nano‐LC‐MS/MS Analyzes

#### 2.6.1 Data‐Dependent Acquisition (DDA) Library Construction

Peptides were dissolved in mobile phase A (as above), centrifuged (20,000*g*, 10 min), and supernatants analyzed on a Thermo UltiMate 3000 UHPLC system. Following enrichment and desalting with a trap column, samples entered into a self‐loading C18 column (150 μm inner diameter, 1.8 μm particle size, 35 cm length) in which they underwent separation at 500 nL/min as follows: 0–5 min, 5% mobile phase B (as above); 5–90 min, 5%–25% B; 90–100 min, 25%–35% B; 100–108 min, 35%–80% B; 108–113 min, 80% B; 113.5–120 min, 5% B. The column separation was connected directly to a mass spectrometer.

#### 2.6.2 DDA Mass Spectrometry Analyzes

Separated fractions were ionized with the nanoESI source before entry into the Orbitrap Exploris480 tandem mass spectrometer (Thermo Fisher Scientific, MA, USA) for DDA analysis with the following settings: ion voltage = 1.9 kV, primary scan range = 350–1640 m/z, resolution = 120,000, maximum ion implantation time (MIT) = 90 ms; HCD secondary fragmentation mode; fragmentation energy = NCE 30; resolution = 30,000, MIT = automatic mode; dynamic exclusion time = 90 s. The starting m/z for secondary scans was set to automatic mode. Precursor screening conditions for secondary fragmentation range from a charge of 2 + to 6 + , with peak intensity exceeding 2E4 in the top 30 precursor ions. Automatic gain control (AGC) is set to: Level 1 300%, Level 2 100%.

#### 2.6.3 Data‐Independent Acquisition (DIA) Sample Analyzes

Peptides were reconstituted and centrifuged as mentioned above using the same mobile phase A and injected for analysis with a Thermo UltiMate 3000 UHPLC system (150 μm inner diameter, 1.8 μm particle size, 35 cm length) in which they underwent separation at 500 nL/min with the same mobile phase B using the following linear gradient settings: 0–5 min, 5% B; 5–45 min, 5%–25% B; 45–50 min, 25%–35% B; 50–52 min, 35%–80% B; 52–54 min, 80% B; 54.5–65 min, 5% B. The liquid phase separation of the column was connected directly to a mass spectrometer.

#### 2.6.4 DIA Mass Spectrometry Analyzes

Separated fractions were ionized with the nano‐electrospray ionization (nanoESI) source before entry into the Orbitrap Exploris 480 tandem mass spectrometer for DIA with the following settings: ion source voltage = 1.9 kilovolts (kV), primary scanning range = 400–1250 mass‐to‐charge ratio (m/z); resolution = 120,000; maximum injection time (MIT) = 90 ms (ms). The 400–1250 m/z range was divided into 50 windows to enable continuous window fragmentation and signal acquisition. The ion fragmentation mode was set to higher‐energy collisional dissociation (HCD), with a normalized collision energy (NCE) of 30, MIT in automatic mode, and fragment ion detection with a resolution of 30,000. Automatic gain control (AGC) was set to Levels 1 (300%) and 2 (1000%).

### 2.7 DIA Data Analyzes

DIA data were corrected based on indexed retention time (iRT) peptide retention time values, and the sequential window acquisition of all theoretical mass spectra (SWATH‐MS) target‐decoy model was then used to assess data at a 1% false discovery rate (FDR) threshold to yield significant quantitative results.

### 2.8 Gene Ontology (GO) Enrichment Analyzes

GO enrichment analyzes can provide insight into annotated GO terms significantly enriched in a given set of proteins or genes of interest relative to the background of all quantified proteins or genes for a given species, thereby providing insight into the functional roles played by these genes or proteins. Genes or proteins are initially mapped to the GO database (http://www.geneontology.org/), counting the number of targets for each term and then employing hypergeometric tests to identify GO terms in which the genes or proteins are significantly enriched relative to the overall protein/gene background. The formula used for such testing is as follows:

$$p=1-\sum_{j-0}^{m-1}\frac{\left(\begin{array}{l} M\\ j \end{array}\right)\left(\begin{array}{l} N-M\\ n-j \end{array}\right)}{\left(\begin{array}{l} N\\ n \end{array}\right)},$$

where *N* represents the number of genes/proteins with GO annotations of all genes/proteins for a given species, while n represents the number of genes or proteins of interest in *N*, *M* denotes the number of genes/proteins annotated to a given GO term as a fraction of all genes/proteins, and m indicates the number of genes/proteins of interest in a given GO term. *p* ≤ 0.05 was selected as the significance threshold, with those terms below this threshold being considered significantly enriched in the genes/proteins of interest, therefore offering insight into the biological functions played by these genes/proteins.

### 2.9 Validated on a Variety of Microarray Platforms

External datasets were accessed from the Gene Expression Omnibus (GEO) database (https://www.ncbi.nlm.nih.gov/geo/), including platforms GPL27951 (GSE142615) and GPL24247 (GSE229925, GSE171546). Data from the same platform were normalized using the “sva” R package before model validation to ensure consistency.

### 2.10 MSstats Significance Analyzes

Male C57BL/6 mice, aged 8–10 weeks and weighing between 22 and 25 g, were obtained from (Shanghai Slake Experimental Animal Co.) Ltd and housed under standard environmental conditions, including a temperature of 22  ±  2°C, a 12 h light/dark cycle, and ad libitum access to food and water. All experimental procedures were conducted in accordance with the guidelines of the Animal Experimentation Ethics Committee of Taizhou College, with appropriate ethical approvals obtained (Approval No. tzy‐2023125). Septic cardiomyopathy was induced using the cecal ligation and puncture (CLP) method. Briefly, mice were anesthetized with 3% isoflurane to ensure complete unresponsiveness and positioned in the supine position under aseptic conditions. A midline abdominal incision was made to expose the cecum, which was then ligated approximately 1 cm proximal to the distal end of the bowel and punctured once with a 30‐gauge needle to release fecal matter and initiate infection. The surgical site was covered with a sterile dressing to prevent infection. Control mice underwent a sham procedure involving only the abdominal incision and suturing without ligation or puncture. Forty‐eight hours after the CLP or sham procedures, blood samples were collected from each mouse via cardiac puncture for subsequent analyzes.

### 2.11 Western Blot

Plasma from mice subjected to the septic cardiomyopathy model was lysed using RIPA lysis buffer supplemented with protease and phosphatase inhibitors. Total proteins were extracted through ultrasonic fragmentation followed by centrifugation at 12,000 rpm for 15 min at 4°C. Protein concentration was determined using the BCA protein quantification kit. An aliquot of 20–30 µg of the protein sample was mixed with 5× loading buffer and denatured by heating at 95°C for 5 min. The denatured proteins were then separated by electrophoresis on a 10% SDS‐PAGE gel (100 V for 1 h) and subsequently transferred to a PVDF membrane via wet transfer (100 V for 1 h). Following transfer, the membranes were blocked in TBST containing 5% skimmed milk powder for 1 h to prevent nonspecific binding. The membranes were incubated overnight at 4°C with a rabbit anti‐IL7B (Proteintech Group Inc) primary antibody diluted 1:1000. On the following day, the membranes were washed three times with TBST for 5 min each and then incubated with an HRP‐conjugated anti‐rabbit IgG secondary antibody diluted 1:5000 for 1 h at room temperature. After additional washes, protein bands were visualized using an enhanced chemiluminescence (ECL) detection system, and images were captured with a chemiluminescent imaging system (Bio‐Rad ChemiDoc). The gray values of the IL7B bands were quantified using ImageJ software and normalized to an internal reference protein (β‐Actin), to ensure accurate quantitative analysis.

### 2.12 RNA Isolation and Quantitative Real‐Time PCR (qRT‐PCR)

Total RNA was isolated from frozen left ventricular tissue or cultured cells using TRIzol reagent following the manufacturer's instructions. RNA concentration and purity were assessed using a NanoDrop spectrophotometer (Thermo Fisher Scientific). One microgram (1 µg) of purified RNA was reverse‐transcribed into complementary DNA (cDNA) using the Omniscript Reverse Transcription Kit (Vazyme Biotech, Nanjing, China) and random hexamer primers according to the manufacturer's protocol. Quantitative real‐time PCR (qRT‐PCR) was performed using SYBR Green Master Mix on a real‐time PCR system. The amplification protocol consisted of an initial denaturation step at 95°C for 10 s, followed by 40 cycles of denaturation at 95°C for 10 s, annealing at 60°C for 20 s, and extension at 72°C for 15 s. The relative mRNA expression levels of IL27B were calculated using the ΔΔCt method, normalized to the endogenous reference gene GAPDH. The primer sequences used in this study are listed below:

IL27B_F: GTCGTCACTTGAGGGTGGC

IL27B_R: CAAAGTTCACAAAGCCCCGC

GAPDH_F: GAAGGTGAAGGTCGGAGTC

GAPDH_R: GAAGATGGTGATGGGATTTC

### 2.13 MSstats Significance Analyzes

The R package MSstats [23] in Bioconductor enables the detection of significant differences in peptides or proteins between samples. It employs a core algorithm based on linear mixed‐effects models and is commonly employed in targeted proteomics, MRM, nonstandard quantification, and Sequential Window Acquisition of All Theoretical Mass Spectra (SWATH) quantitative experiments. Msstats analyzes entail preprocessing data using a set comparison group and then performing significance testing based on the resultant model. Differential protein expression was then detected based on a fold‐change > 1.5 and *p* < 0.05. Differentially expressed proteins were also used for enrichment analyzes.

## 3 Results

### 3.1 Proteomic Analysis in Patients With SCM

**Figure 1:**
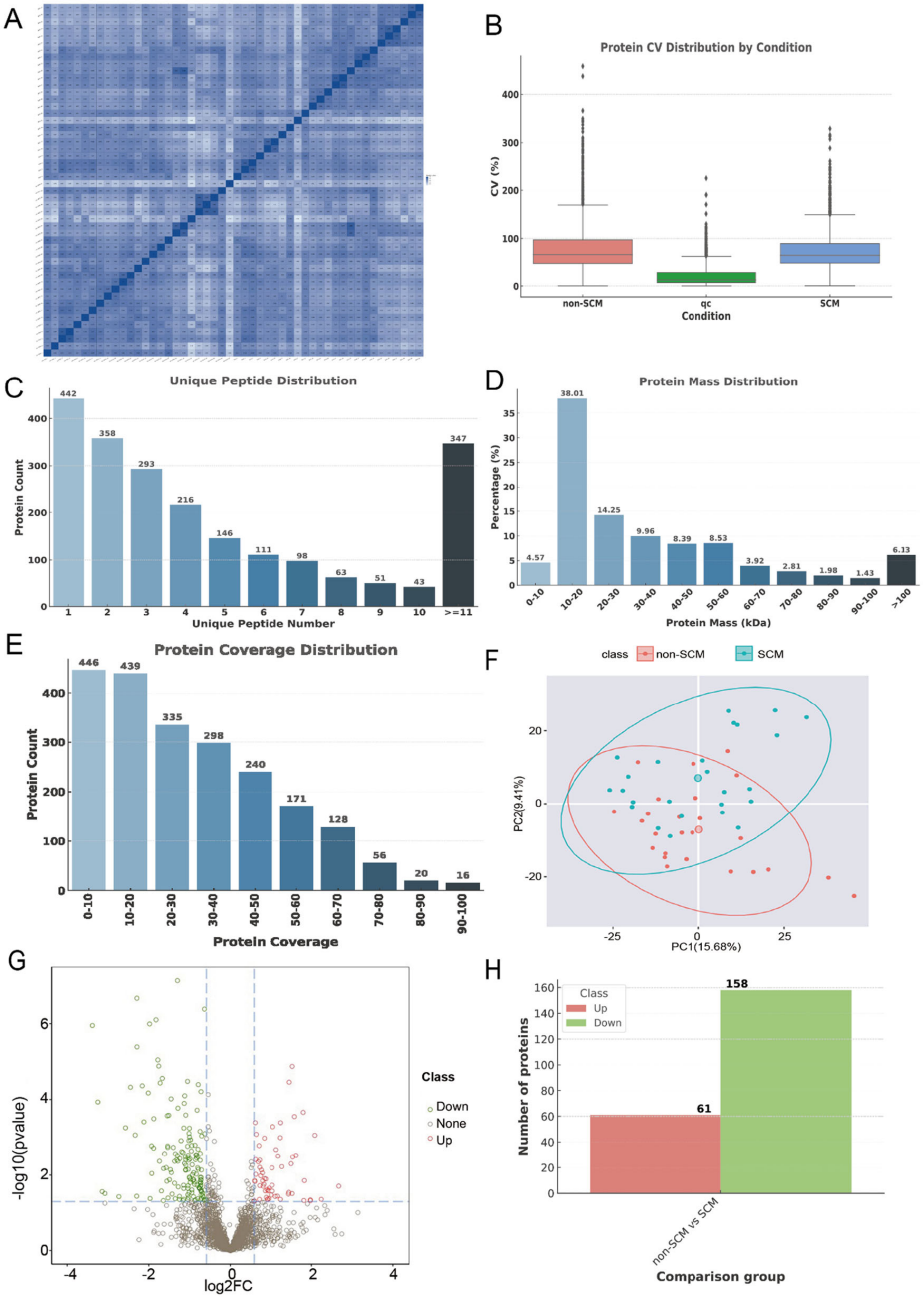
Proteome blood analysis of septic cardiomyopathy. (A) Heat map of sample correlation analysis. Both the X and Y axes represent samples, with color intensity indicating the correlation coefficient. Darker colors represent a higher correlation, while lighter colors represent a lower correlation. (B) Coefficient of variation (CV) distribution. The X‐axis denotes the sample groups, and the Y‐axis represents the corresponding CV. In large projects, a quality control (QC) group is used to evaluate the stability and repeatability of the experiment. (C–E) Protein identification analysis. (C) Unique peptide distribution. (D) Protein mass distribution. (E) Protein coverage distribution. (F–H) Quantitative differential analysis. (F) Principal component analysis (PCA). The X‐axis represents the first principal component, and the Y‐axis represents the second principal component, showing the separation between groups based on proteomic data. (G) Volcano plot of differentially expressed proteins. The X‐axis represents protein fold change (log2), and the Y‐axis represents the corresponding ‐log10 (*p*‐value). Red dots indicate significantly upregulated proteins, green dots indicate significantly downregulated proteins and gray dots represent proteins with no significant change. (H) Bar chart of differentially expressed proteins. There are 61 upregulated proteins and 158 downregulated proteins.

To guarantee the reliability of the data, a series of quality control measures were implemented, including assessments of within‐group coefficient of variation (CV), principal component analysis (PCA), and quantitative correlation evaluations. Mixed samples, prepared by pooling all individual samples, were periodically analyzed as quality control (QC) samples to monitor the stability and reproducibility of the data (Figure 1A). The consistency of protein quantification across samples was further assessed using Pearson correlation coefficients (Figure 1B).

Proteomic analysis was conducted using the Orbitrap Exploris 480 mass spectrometer (Thermo Fisher Scientific) in DIA mode. A total of 50 samples were analyzed, leading to the identification of 25,062 peptides and 2168 proteins. The identified proteins were characterized based on their unique peptide composition, protein mass distribution, and protein coverage (Figure 1C–E).

PCA revealed distinct clustering patterns between SCM and non‐SCM sepsis patients, reflecting significant differences in their proteomic profiles (Figure 1F). Differential protein expression was analyzed using the Benjamini–Hochberg correction, identifying 216 proteins with significant changes, of which 61 were upregulated and 158 were downregulated (Fold change > 1.5, *p* < 0.05) (Figure 1G,H).

### 3.2 Relationships Between Proteomic and Transcriptomic Data

**Figure 2:**
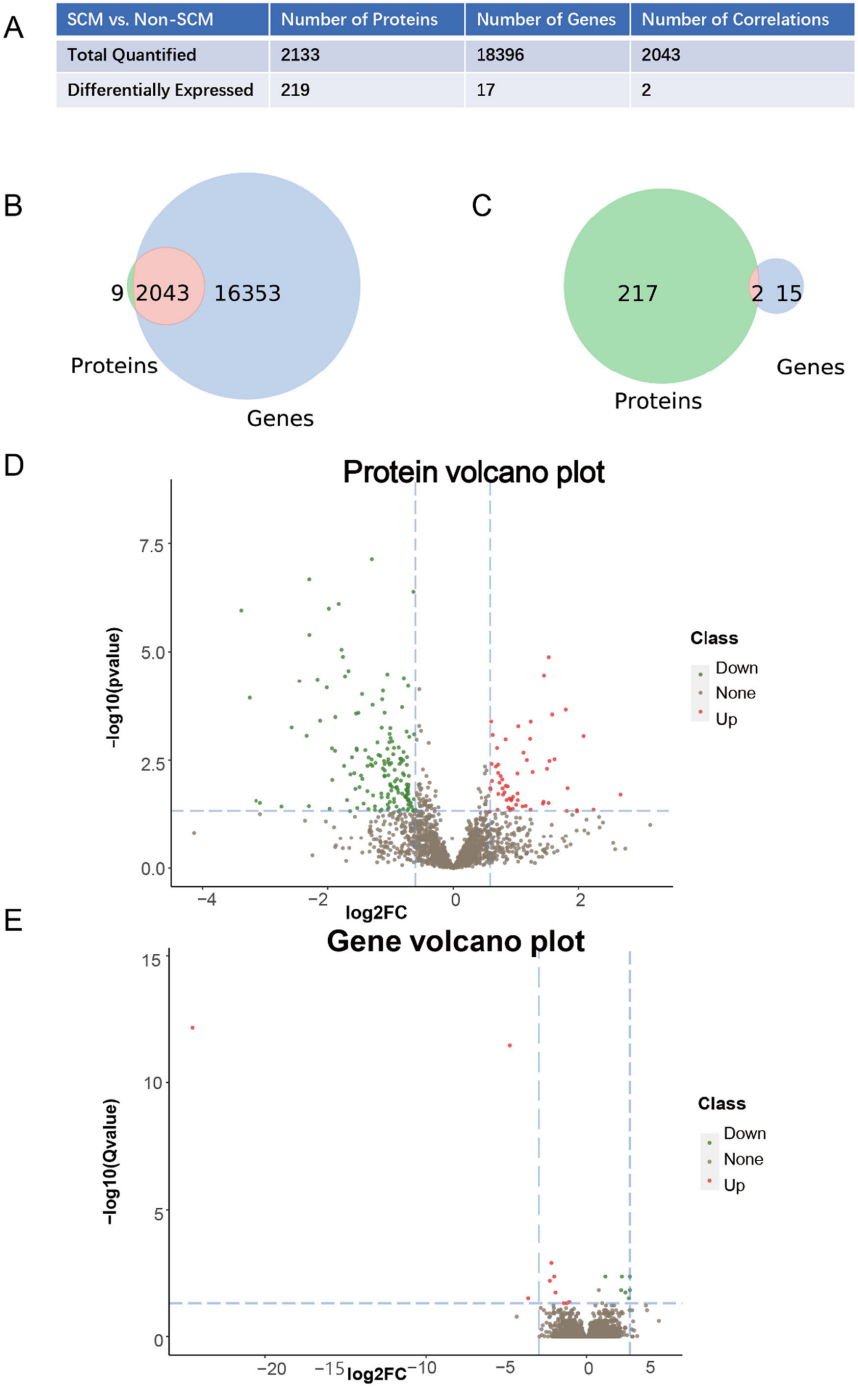
Number association analysis of proteome and transcriptome data. (A) Analysis of the relationship between the number of proteins and genes that are correlated in both quantification and significance. (B) Venn diagram showing the correlation between all quantified proteome and transcriptome data. The light red circle represents the number of entities correlated between the two omics, the light blue circle represents genes specific to the transcriptome, and the light green circle represents proteins specific to the proteome. (C) Venn diagram of the correlation between differentially expressed proteins and genes at the quantitative level. The light red circle indicates the number of entities with correlated differential expression in both omics, the light blue circle represents differentially expressed genes specific to the transcriptome, and the light green circle represents differentially expressed proteins specific to the proteome. (D) Volcano plot of the proteome, where red dots indicate upregulated proteins, green dots represent downregulated proteins, and gray dots signify non‐differential proteins. (E) Volcano plot of the transcriptome, with red dots indicating upregulated genes, green dots representing downregulated genes, and gray dots indicating non‐differential genes.

To explore the relationships between the proteomic and transcriptomic datasets, association analyzes were conducted, identifying a total of 2043 genes and proteins with significant correlations between the two datasets. Differential expression analysis (criteria: log2FC > 1.5, adjusted *p* < 0.05) revealed two overlapping targets shared by both datasets (Figure 2A–C). Further analysis showed that 61 proteins were upregulated and 158 proteins were downregulated at the proteomic level. Similarly, 10 genes were upregulated and 7 genes were downregulated at the transcriptomic level (Figure 2D,E). These results highlight the integration of proteomic and transcriptomic data to uncover overlapping and distinct molecular signatures, providing insights into the complex regulatory mechanisms at play.

### 3.3 Proteome and Transcriptome Expression Correlation Analysis

**Figure 3:**
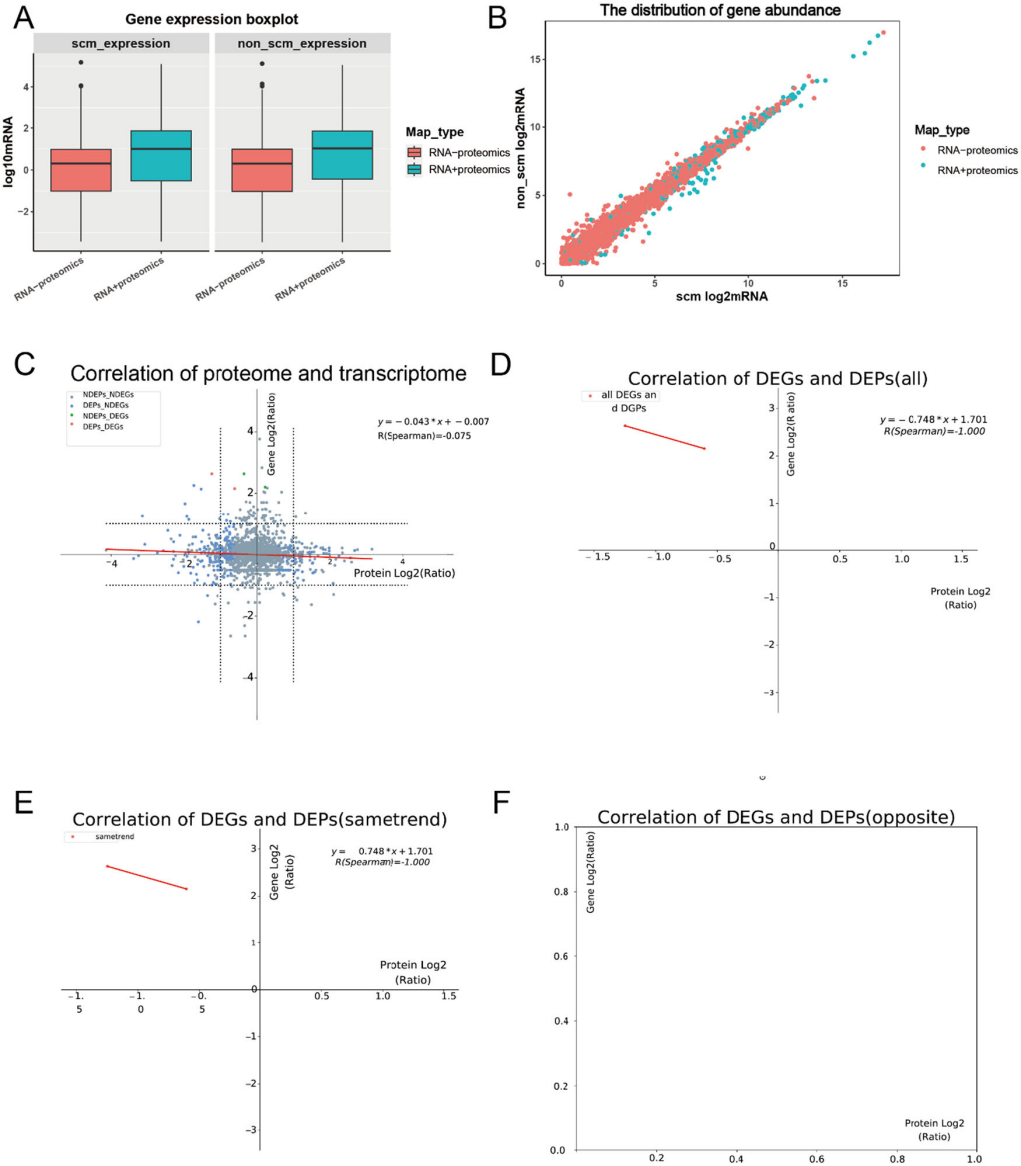
Correlation analysis between proteome and transcriptome expression levels. (A) Boxplot: The distribution of gene abundance correlated (green) and uncorrelated (red) with proteome data. The Y‐axis represents gene expression levels. (B) Scatterplot: The X‐axis represents gene expression levels in the experimental group, and the Y‐axis represents gene expression levels in the control group. Green dots indicate genes correlated with proteome data, while red dots represent uncorrelated genes. (C) Expression correlation plot: The X‐axis represents protein expression levels, and the Y‐axis represents gene expression levels. Gray dots: No significant differences in either mRNA or protein levels. Blue dots: Significant differences in protein expression only. Green dots: Significant differences in gene expression only. Red dots: Significant differences at both mRNA and protein levels. (D) Differential expression correlation plot: This plot focuses on significantly differentially expressed proteins and genes. The X‐axis represents protein expression levels, and the Y‐axis represents gene expression levels. (E) Correlation plot for same expression trends: The X‐axis shows protein expression levels, and the Y‐axis shows gene expression levels for targets with consistent up‐ or downregulation trends. (F) Correlation plot for opposite expression trends: The X‐axis represents protein expression levels, and the Y‐axis represents gene expression levels for targets with contrasting expression trends.

Based on transcriptomic data, genes were categorized as associated or nonassociated, and their expression levels were visualized using boxplots and scatterplots (Figure 3A,B). The relationship between mRNA and protein expression levels was classified into five distinct groups: DEPs_DEGs_SameTrend: Targets that were upregulated or downregulated consistently at both the mRNA and protein levels; DEPs_DEGs_Opposite: Targets with opposing differential expression between mRNA and protein levels; DEPs_NDEGs: Proteins that were differentially expressed but showed no differential expression at the mRNA level; NDEPs_DEGs: Genes that were differentially expressed but not at the protein level; NDEPs_NDEGs: Targets that showed no differential expression at either the mRNA or protein level (Figure 3C). Spearman correlation analysis identified two significantly downregulated targets at both the gene and protein levels: IL‐27B and carbonic anhydrase, with a perfect negative correlation (R = −1.00) (Figure 3D,E). Notably, no upregulated targets demonstrated strong correlations between mRNA and protein expression levels (Figure 3F).

These findings highlight the complex regulatory relationships between transcriptomic and proteomic changes, emphasizing the importance of integrating multi‐omics data to fully understand the molecular mechanisms underlying the observed biological processes.

### 3.4 GO Enrichment Analyzes

**Figure 4:**
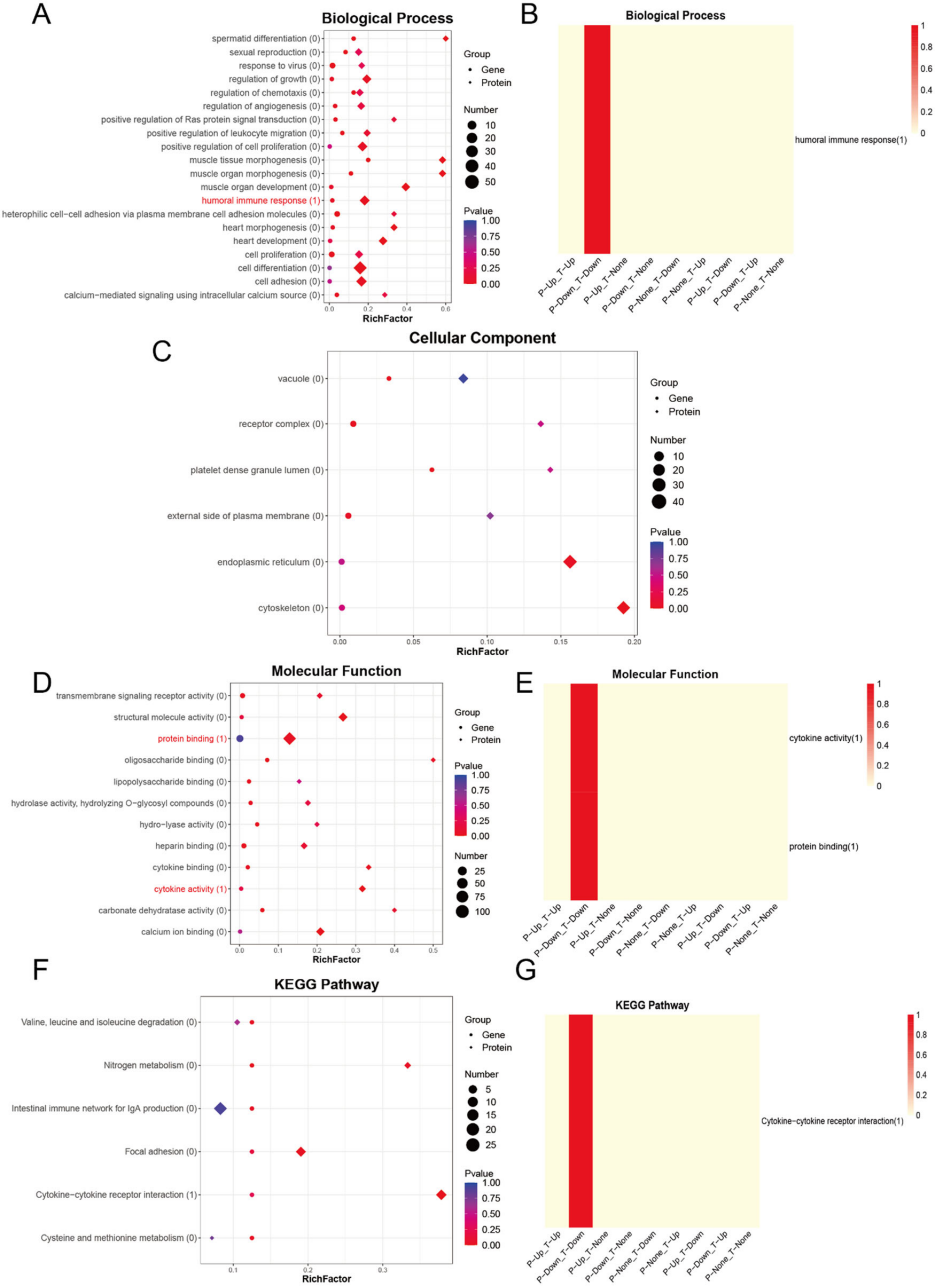
Enrichment association analysis of omics data. (A, C, D, F) Bubble plots show enrichment of differentially expressed genes and proteins across GO and KEGG terms. The Y‐axis lists terms with the number of associated molecules in parentheses, and the X‐axis indicates the RichFactor. Rhombuses and circles represent transcriptomic and proteomic data, respectively. Bubble size reflects the number of enriched molecules, and color indicates statistical significance (*p*‐value).(B, E, G) Heatmaps display pathway involvement across nine omics expression patterns (T: transcriptome; P: proteome; Up/Down: up‐/downregulated; None: unchanged). Color intensity reflects the proportion of molecules involved in each pathway under different conditions. GO analysis revealed significant enrichment in immune response, cell proliferation, and cytokine‐related functions. KEGG pathways such as *cytokine–cytokine receptor interaction* and *IgA immune network* were prominently enriched, highlighting immune regulation as a key feature across omics levels.

GO enrichment analyzes were performed to systematically categorize genes and proteins into the three primary categories: molecular function (MF), cellular component (CC), and biological process (BP) (Figure 4A,C,D). At the molecular function level, IL‐27B was found to be significantly associated with cytokine activity and protein binding, suggesting its potential role in mediating immune signaling and molecular interactions (Figure 4C). In the biological process category, IL‐27B was notably involved in the humoral immune response, with both its gene and protein expression levels demonstrating significant downregulation, indicating a potential dampening effect on this critical immune pathway (Figure 4B).

**Figure 5:**
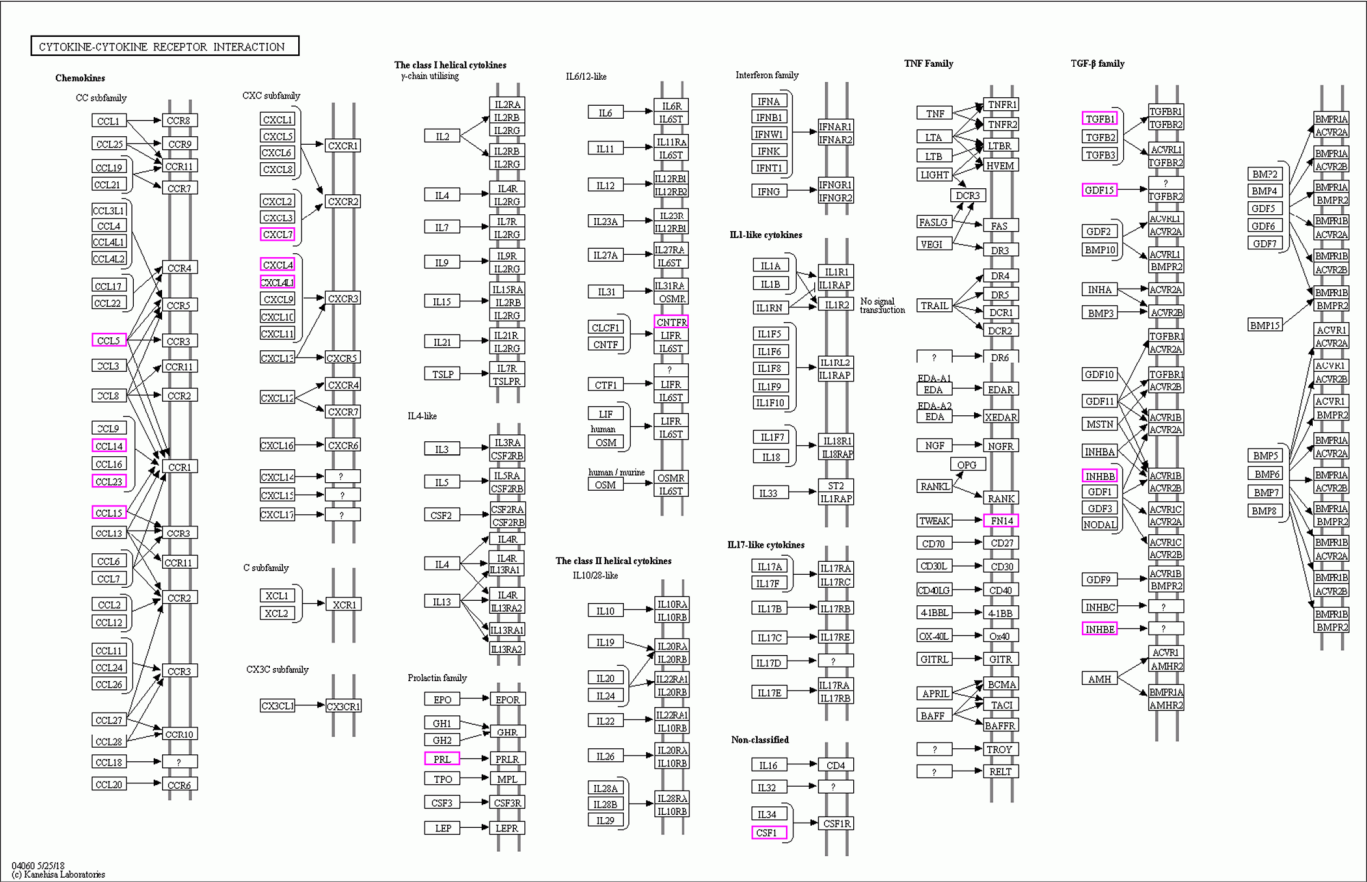
Integrated analysis of metabolic pathways of differential proteins and genes.as genes. As shown in the figure, CNTFR and IL‐27B are involved in the IL‐6/IL‐12‐like signaling pathway.

To further elucidate the roles of differentially expressed genes and proteins, pathway analyzes were conducted using the KEGG database. IL‐27B was identified as a key participant in the cytokine‐cytokine receptor interaction pathway, a central signaling axis that regulates immune responses and intercellular communication. Both gene and protein expression levels of IL‐27B were consistently downregulated in this pathway, reinforcing its potential regulatory role in immune modulation (Figure 4E,F). Additionally, annotation of the KEGG pathways associated with differentially expressed genes and proteins revealed that IL‐27B functions in the cytokine‐cytokine receptor interaction pathway through the IL‐6/IL‐12‐like signaling pathway (Figure 5).

These findings not only highlight the functional roles of IL‐27B in cytokine‐mediated immune responses but also underscore its broader impact on metabolic and immune regulatory networks. The integration of GO and KEGG analyzes provides a comprehensive understanding of the molecular mechanisms underlying IL‐27B's involvement in the progression of SCM, offering valuable insights for future research into its potential as a therapeutic target.

### 3.5 EBI3 Cross‐Platform Validation

**Figure 6:**
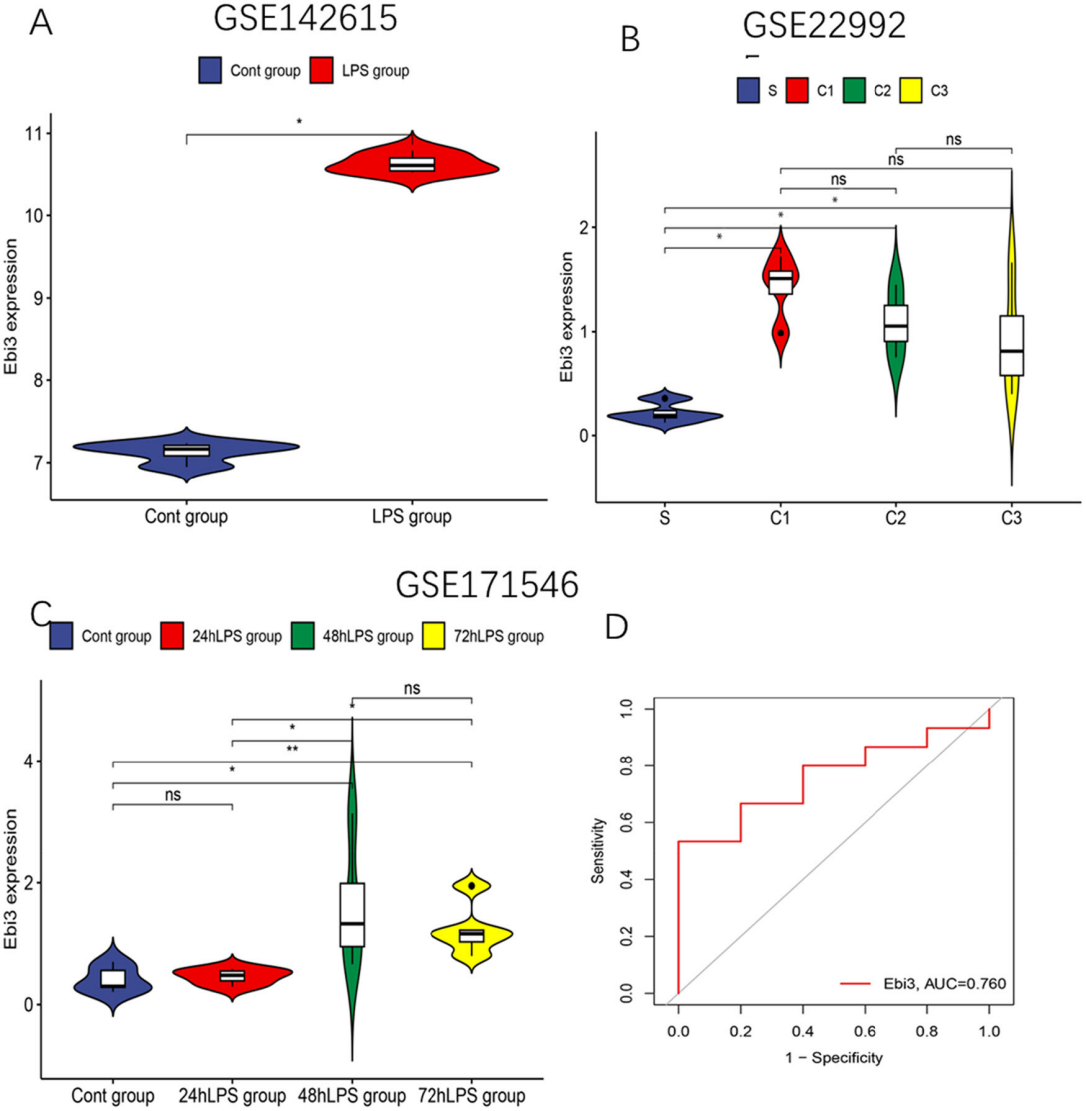
Analysis of EBI3 expression in septic cardiomyopathy (SCM) using GEO datasets GSE142615, GSE229925, and GSE171546. (A) In the GSE142615 data set, a significant difference in EBI3 expression was observed between patients with SCM and those with sepsis alone (*p* < 0.05), indicating a distinct expression pattern associated with SCM. (B) The GSE229925 data set revealed differential EBI3 expression across various SCM subgroups classified by ejection fraction: high ejection fraction (HEF, LVEF ≥ 90%) (C1), low ejection fraction (LEF, LVEF < 65%) (C2), and normal ejection fraction (NEF, 65% ≤ LVEF < 90%) (C3). Significant differences in EBI3 expression were found between these groups (*p* < 0.05). (C and D) Although the early‐stage SCM group (24hLPS) did not exhibit statistically significant changes in EBI3 expression, a significant increase in EBI3 levels was observed in the 48 h LPS and 72 h LPS groups as SCM progressed (*p* < 0.05).

IL27B, also known as EBI3, is the β subunit of the IL27 cytokine complex. To validate the expression of the EBI3 gene across platforms, we analyzed three GEO datasets: GSE142615, GSE229925, and GSE171546 (Figure 6A). In the GSE142615 data set, comparing sepsis patients (control group) to SCM patients (experimental group) revealed a significant difference in EBI3 expression (*p* < 0.05) (Figure 6B). For GSE229925, which categorized sepsis patients into groups based on LVEF—high LVEF (HEF, LVEF ≥ 90%), low LVEF (LEF, LVEF < 65%), normal LVEF (NEF, 65% ≤ LVEF < 90%), and sepsis controls—significant differences in EBI3 expression were observed (*p* > 0.05) (Figure 6C,D). In the GSE171546 data set, no significant differences in EBI3 expression were detected between septic rats and those with SCM within the first 24 h; however, significant differences were observed at 48 and 72 h. The area under the curve (AUC) for GSE171546 was 0.760, exceeding the threshold of 0.6, indicating strong predictive capability. These findings demonstrate that EBI3 expression varies across different conditions and time points, suggesting its potential as a diagnostic or predictive marker for SCM.

### 3.6 qPCR and Western Blot Validation

**Figure 7:**
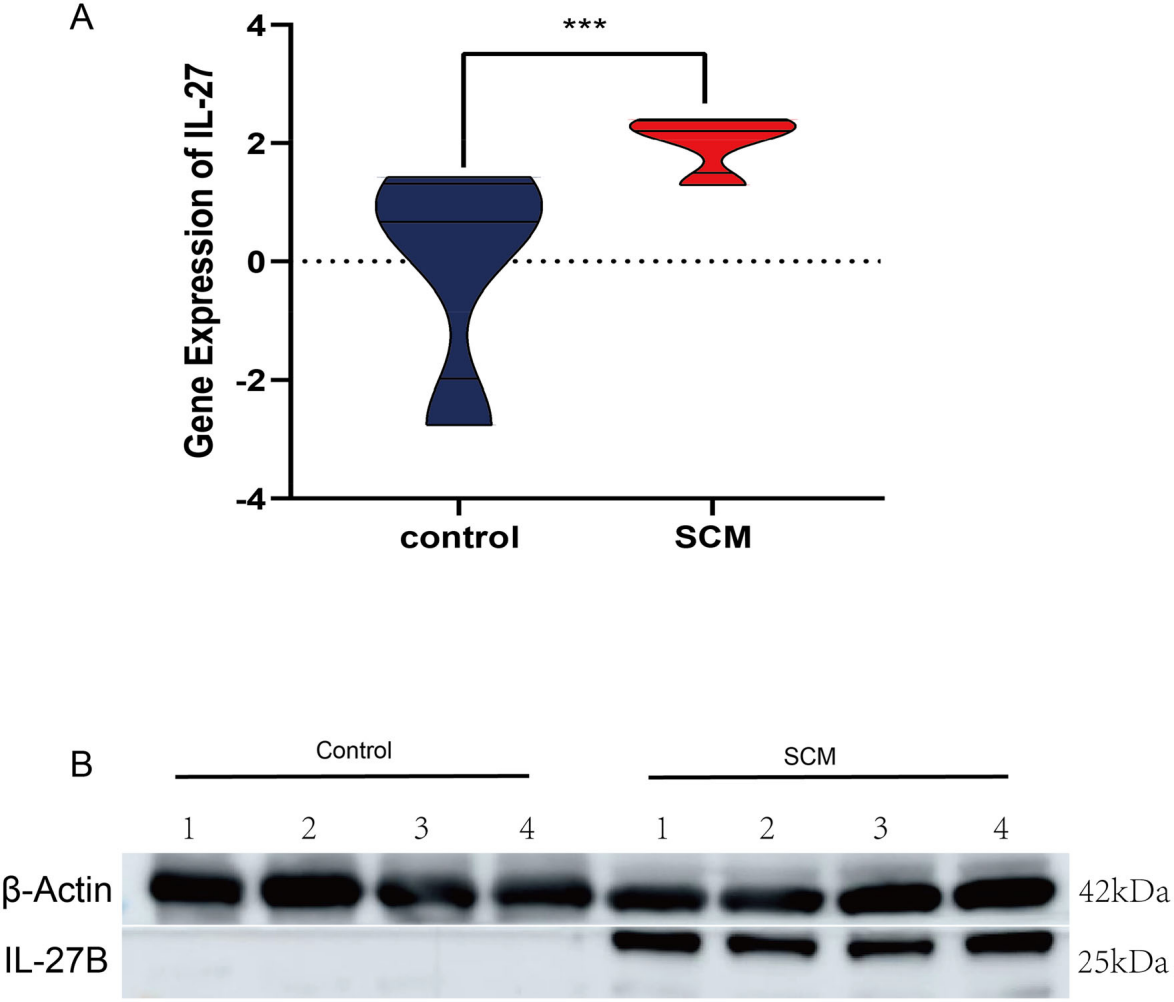
qPCR and Western Blot Validation. (A) Violin plot comparing the gene expression levels of IL‐27 between control and SCM groups. The data shows a significant upregulation of IL‐27 in the SCM group compared to the control (***p < 0.001); (B) Western blot analysis of IL‐27B protein levels in control and SCM groups. β‐Actin is used as a loading control. Molecular weights for IL‐27B (~25 kDa) and β‐Actin (~42 kDa) are indicated on the right side of the blot.

We used qPCR to validate the expression levels of two genes identified in the omics analysis. The results showed that IL‐27B expression was significantly higher in the SCM mouse model compared to controls (Figure 7A). To confirm these changes at the protein level, we performed Western blot analysis (Figure 7B). Consistent with the qPCR results, the Western blot showed a significant increase in IL‐27B protein expression in the SCM group.

## 4 Discussion

SCM is a form of cardiomyopathy characterized by intrinsic diastolic and contractile dysfunction of both sides of the heart in patients suffering from sepsis [8]. Early detection of myocardial dysfunction is essential for effectively managing affected patients; however, no reliable biomarkers currently exist for assessing SCM [1]. Elucidating the underlying pathological mechanisms of SCM could help in early detection and may also identify novel therapeutic targets specific to the heart, ultimately improving patient outcomes. In this study, proteomic analyzes of serum samples from 50 patients were used to investigate the protein‐level changes associated with the progression from sepsis to SCM. Integrating multi‐omics data established a robust biological network related to the onset of SCM, providing insight into the mRNA and protein‐level changes characteristic of this condition.

Differential expression analysis of proteins and mRNA transcripts between SCM and non‐SCM patients revealed two consistently differentially expressed targets: IL‐27B and carbonic anhydrase. GO enrichment analyzes indicated that IL‐27B was associated with cytokine activity, protein binding, and humoral immune response. Cytokine activity plays a crucial role in regulating immune responses and directing immune cell trafficking within the body [24, 25]. The association between humoral immunity and SCM has been previously established [26], further supporting the relevance of our findings. KEGG pathway analyzes indicated that IL‐27B was involved in the cytokine‐cytokine receptor interaction pathway, suggesting that its downregulation may be associated with SCM progression, likely through the modulation of immune and inflammatory responses.

Here, blood samples were used for both proteomic and transcriptomic analyzes, with the resultant multi‐omic data set offering greater insight than either data set in isolation. The identification of correlations between methodologies and datasets can also enable the construction of more robust, functionally validated datasets, revealing disease pathology‐related changes spanning from the mRNA to the protein level. The observed changes can potentially be used as biomarkers for disease diagnosis of targets for therapeutic intervention. SCM‐related biomarker identification can improve disease diagnosis while offering new molecular insight into the etiology of this disease. In particular, our study identified IL‐27B as a potential marker for SCM incidence, which could be useful in clinical diagnostics.

Members of the Interleukin‐6 and Interleukin‐12 (IL‐6/IL‐12) cytokine family are known to function as key regulators of innate and adaptive immunity, consisting of α and β subunits, thus distinguishing them from most cytokines [27]. As a member of this family, IL‐27 serves as a regulator of inflammatory activity. IL‐27 consists of an α subunit and a β subunit (IL27B, EBI3), which are vital in antiviral and immunomodulatory contexts [28, 29, 30]. IL‐27 can influence humoral immunity by affecting T follicular helper cells, which produce high‐affinity antibodies from maturing B cells [31, 32, 33]. The role of IL‐27 in sepsis has been suggested by previous studies [34, 35], and Feng Gaof et al. have also highlighted its importance in SCM progression [36]. The signaling activity of IL‐27 through the IL‐27B (EBI3) receptor protein influences various immune cells, potentially contributing to the immune dysregulation seen in sepsis and SCM [37, 38, 39]. Here, joint proteomics and transcriptomic analyzes revealed that IL‐27B was differentially expressed between sepsis patients with and without SCM. The expression of IL‐27B affects the development of SCM and could thus be used as a potential diagnostic marker and therapeutic target for SCM.

Recent studies have identified IL‐6 and IL‐12 as two critical cytokines closely associated with M1 macrophage polarization. M1‐polarized macrophages are a subset of macrophages characterized by their proinflammatory, antimicrobial, and antitumor functions [40]. They play a pivotal role in immune responses, particularly in combating bacterial and viral infections [41]. Research indicates that M1 macrophage polarization can promote the progression of spontaneous coronary atherosclerosis [42]. Based on these findings, we hypothesize that IL27B may induce M1 macrophage polarization, thereby facilitating the advancement of SCM.

Recent findings have highlighted the essential role of mitochondria in myocardial injury. Mitochondrial dysfunction is closely linked to the structural and functional regulation of myocardial endothelial cells, making it a major contributor to cardiovascular diseases [43]. Dysfunctional mitochondria disrupt energy metabolism, increase oxidative stress, and trigger apoptosis. In ischemia‐reperfusion (I/R) injury, the opening of the mitochondrial permeability transition pore (MPTP) leads to calcium overload and increased production of reactive oxygen species (ROS), which induce cardiomyocyte death. Additionally, damage to mitochondrial DNA (mtDNA) activates inflammatory responses, further exacerbating myocardial injury, while imbalances in mitochondrial dynamics result in energy insufficiency and cell death [44, 45]

Furthermore, M1‐polarized macrophages undergo mitochondrial metabolic reprogramming, favoring aerobic glycolysis to rapidly meet energy demands while inhibiting oxidative phosphorylation and reducing fatty acid oxidation. During this process, mitochondria generate substantial amounts of ROS, which not only directly eliminate pathogens but also activate proinflammatory gene expression through ROS‐mediated signaling pathways [46, 47]. Disruption of mitochondrial metabolism leads to the accumulation of metabolites such as succinate, which stabilizes hypoxia‐inducible factor‐1α (HIF‐1α), further promoting M1 polarization and inflammatory responses. Additionally, the release of mtDNA can activate inflammatory pathways, enhancing immune responses [48]. Therefore, mitochondria play a crucial role in the energy metabolism, signal transduction, and functional regulation of M1 macrophages, supporting their proinflammatory and antimicrobial activities. IL27B enhances the proinflammatory and antimicrobial functions of M1‐polarized macrophages by regulating mitochondrial metabolism and signaling, thereby playing a significant role in immune responses and inflammation regulation.

Our cross‐platform validation using datasets from GEO (GSE142615, GSE229925, and GSE171546) revealed a significant trend in EBI3 gene expression. EBI3 expression was significantly elevated in SCM compared to sepsis, suggesting its involvement in the progression from sepsis to SCM. Our examination of GSE229925 revealed consistently increased levels of EBI3 expression across various subtypes of SCM. However, EBI3 expression levels did not differ significantly across LVEF subtypes of SCM (HEF, LEF, NEF). This finding implies that EBI3 may not significantly contribute to differentiating SCM subtypes based on LVEF. EBI3 expression levels increased with the progression of SCM, highlighting EBI3's potential role in driving SCM pathology.

This study provides novel insights into the pathophysiological mechanisms underlying SCM by identifying IL‐27B as a potential biomarker for early diagnosis and as a therapeutic target. The multiomic approach used here not only enhances our understanding of the molecular events driving SCM but also opens up avenues for the development of new diagnostic tools that can detect myocardial abnormalities at earlier stages of sepsis progression. As early intervention is crucial in improving patient outcomes, the identification of IL‐27B could lead to the creation of predictive biomarkers that guide clinical decision‐making. Additionally, the proteomic analysis conducted by Kexin Cai et al. on an SCM rat model revealed that the complement and coagulation cascades play a critical role in disease progression [49]. Similarly, Timothy P. Fitzgibbons et al. observed that the unexpected and profound activation of inflammatory and prothrombotic pathways significantly increases the risk of mortality associated with this condition. In our study [50]. We found that IL‐27B influences SCM progression through the IL‐6/IL‐12‐like pathway within the cytokine‐cytokine receptor interaction pathway, which plays a critical role in regulating inflammation and immune responses. These findings offer a new perspective for exploring and understanding the mechanisms underlying SCM progression, providing valuable insights into the pathophysiology of the disease.

In summary, IL‐27B may influence myocardial cell injury and promote the progression of SCM by regulating mitochondrial function and promoting M1‐polarized macrophages. Based on these findings, future research could focus on therapeutic modulation of the IL‐27B signaling pathway to mitigate the progression of SCM and improve cardiovascular outcomes in septic patients. Additionally, this study demonstrates the utility of multiomics analysis, through the integration of proteomics and transcriptomics data, in elucidating the mechanisms of complex diseases. This approach can also be applied to other areas of cardiovascular research. Contributing to the advancement of future medicine, these findings are expected to drive the development of novel therapeutic strategies, promote the progress of precision medicine, and provide new theoretical foundations and practical guidance for the prevention and treatment of cardiovascular diseases, thereby significantly enhancing clinical efficacy and improving patients' quality of life.

## 5 Limitations

This study has several limitations: (1) The sample size was relatively small, comprising only 25 cases each of sepsis and septic cardiomyopathy, all from two medical centers. Larger, multi‐center studies are needed to enhance the credibility and generalizability of the findings; (2) Although proteomic and transcriptomic analyzes were conducted to explore the role of IL‐27B in the progression of SCM, additional evidence is required to validate these findings and assess their broader clinical applicability. Future research incorporating animal models of sepsis and SCM would be valuable for further validation; (3) This study focused exclusively on early‐stage SCM patients, without subgroup analyzes based on the time course or disease severity. This limitation constrains the scope of the findings and their potential implications; (4) The pathogens causing sepsis were not documented during the case collection process, representing a significant limitation. Addressing this in future research will enhance the comprehensiveness and applicability of the results; (5) Variations in the quantity and abundance of proteins in blood samples may have limited the detection of low‐abundance proteins. This constraint could result in discrepancies between proteomic and transcriptomic analyzes, potentially leading to the omission of certain genes in Venn diagram analyzes. (6) Currently, there is a lack of experimental evidence confirming the interactions between IL‐27B, M1‐polarized macrophages, and mitochondria. Future studies should aim to validate these associations through both in vitro and in vivo experiments to further investigate the specific mechanisms by which IL‐27B regulates macrophage polarization and mitochondrial function.

## 6 Conclusions

Comprehensive proteomic and transcriptomic analyzes of blood samples from SCM patients revealed differences in IL‐27B expression between sepsis patients with and without SCM. These findings suggest that IL‐27B may serve as a potential biomarker and therapeutic target for SCM. Future research should aim to further elucidate the pathological role of IL‐27B in SCM and confirm its diagnostic and therapeutic value.

## Author Contributions

Yifeng Mao contributed to data curation, investigation, methodology, software, original draft writing, and review and editing. Yongpo Jiang was involved in conceptualization, data curation, and funding acquisition. Xijiang Zhang contributed to investigation, methodology, and validation. Qingqing Chen was responsible for funding acquisition and methodology. Qin Si handled formal analysis, investigation, and visualization. Panpan Xu participated in funding acquisition and investigation. Zhongheng Zhang provided resources, supervision, and support for visualization. Cheng Zheng contributed to funding acquisition, investigation, supervision, and original draft writing. Ronghai Lin was involved in data curation, investigation, methodology, supervision, validation, and visualization.

## Ethics Statement

The authors have nothing to report.

## Consent

We confirm that this study is original and has not been published elsewhere, nor is it currently under consideration for publication elsewhere.

## Conflicts of Interest

The authors declare that the research was conducted in the absence of any commercial or fnancial relationships that could be construed as a potential conflict of interest. Authors claim no competing interests.

## Data Availability

All data in databases will be available to anyone upon request.
